# A new hexa­gonal polymorph of magnesium perchlorate hexa­hydrate obtained from an acetamide medium

**DOI:** 10.1107/S2056989026004871

**Published:** 2026-05-12

**Authors:** Gergana Velyanova, Krasimir Kossev, Rositsa Nikolova

**Affiliations:** a Institute of Mineralogy and Crystallography, Bulgarian Academy of Sciences, Akad. G. Bonchev Str., Bl. 107, 1113 Sofia, Bulgaria; University of Aberdeen, United Kingdom

**Keywords:** crystal structure, hexa­gonal polymorph, magnesium perchlorate hexa­hydrate

## Abstract

A new magnesium perchlorate hexa­hydrate phase, Mg(ClO_4_)_2_·6H_2_O, was obtained from the mixed solvents of water and acetamide. The structure crystallizes in the hexa­gonal space group *P*63*mc* and is isostructural with previously reported *M*(ClO_4_)_2_·6H_2_O (*M* = Zn, Ni, Fe) phases. The Mg site is half occupied, indicating positional disorder of the metal cation. Prolonged crystallization leads to the formation of larger crystals consistent with an expanded unit cell, suggesting the formation of a disorder-related superstructure. The Mg, Cl and one of the O atoms have 3*m*. site symmetry.

## Abstract 2

A new magnesium perchlorate hexa­hydrate phase, Mg(ClO_4_)_2_·6H_2_O, was obtained from the mixed solvents of water and acetamide. The structure crystallizes in the hexa­gonal space group *P*63*mc* and is isostructural with previously reported *M*(ClO_4_)_2_·6H_2_O (*M* = Zn, Ni, Fe) phases. The Mg site is half occupied, indicating positional disorder of the metal cation. Prolonged crystallization leads to the formation of larger crystals consistent with an expanded unit cell, suggesting the formation of a disorder-related superstructure.

## 1. Chemical context

Magnesium perchlorate, Mg(ClO_4_)_2_, forms several hydrated crystalline phases, including the dihydrate, tetra­hydrate and hexa­hydrate, which exhibit structural variability depending on crystallization conditions. Early studies described the hexa­hydrate as ortho­rhom­bic, while later investigations revealed additional structural complexity and possible disorder effects. Previously reported crystal structures of magnesium perchlorate hydrates include ortho­rhom­bic and monoclinic phases (West, 1934 ▸; Robertson & Bish, 2010 ▸; Solovyov, 2012 ▸).

Compounds of the general type *M*(ClO_4_)_2_·6H_2_O (*M* = Zn, Ni, Fe) have been reported to adopt closely related structures characterized by metal-site disorder and partial occupancies (Ghosh & Ray, 1976 ▸; Ghosh *et al.*, 1997 ▸). These studies demonstrate that the disorder of the metal position is an intrinsic feature of this structural family and plays a key role in determining symmetry and phase behavior.

The present structure therefore represents a hexa­gonal polymorph of magnesium perchlorate hexa­hydrate, distinct from previously reported ortho­rhom­bic and monoclinic forms. The compound was obtained from an acetamide-containing medium, indicating that the crystallization environment plays a significant role in directing phase formation in the Mg–ClO_4_–H_2_O system.

## 2. Structural commentary

**Figure 1:**
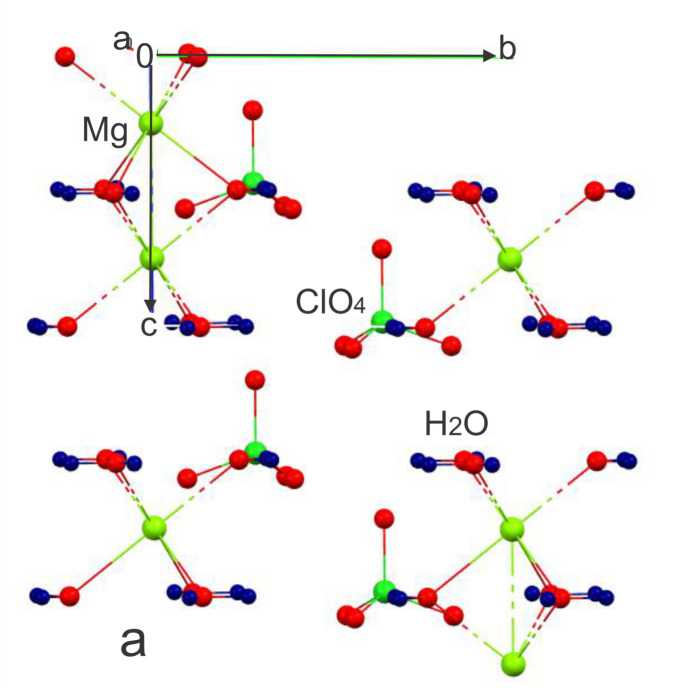
Arrangement of Mg(H_2_O)_6_ octa­hedra and ClO_4_ tetra­hedra in Mg(ClO_4_)_2_·6H_2_O viewed along [100]. Hydrogen bonds are shown as dashed lines.

The title compound, Mg(ClO_4_)_2_·6H_2_O, crystallizes in the hexa­gonal space group *P*63*mc* with *a* = *b* = 7.7942 (3) Å and *c* = 5.2703 (3) Å. The Mg^2+^ cation (site symmetry 3*m*.) is coordinated by six water mol­ecules, forming a slightly distorted octa­hedral [Mg(H_2_O)_6_]^2+^ environment (Fig. 1 ▸). The six Mg—O distances are identical by symmetry at 2.126 (5), while the perchlorate anions retain their usual tetra­hedral geometry with Cl—O distances of 1.426 (5) and 1.428 (3) Å.

The magnesium octa­hedra are connected through shared faces [Mg1⋯Mg1 = 2.63515 (15) Å], forming infinite chains extending along the [001] direction with Mg1—O1—Mg1 = 76.57 (9)°. The half-occupancy of the Mg site implies the presence of disordered inter­ruptions within these chains, resulting in vacant positions along the columns. When this inter­ruption becomes ordered, a lowering of symmetry occurs, leading to ortho­rhom­bic structures (Ghosh *et al.*, 1997 ▸). In contrast, when the disorder is maintained, the structure retains higher symmetry in the hexa­gonal space group. This behavior is consistent with previously reported Zn and Ni perchlorate hexa­hydrate structures (Ghosh & Ray, 1976 ▸), where similar disorder-driven symmetry relationships have been observed.

The perchlorate anions (Cl site symmetry 3*m*.) occupy inter­stitial positions and consolidate the structure through electrostatic inter­actions. During crystallization, large needle-shaped crystals were observed, corresponding to an expanded unit cell of approximately 15 × 15 × 5 Å. This suggests the formation of a superstructure related to partial disorder or long-range ordering effects.

## 3. Supra­molecular features

**Figure 2:**
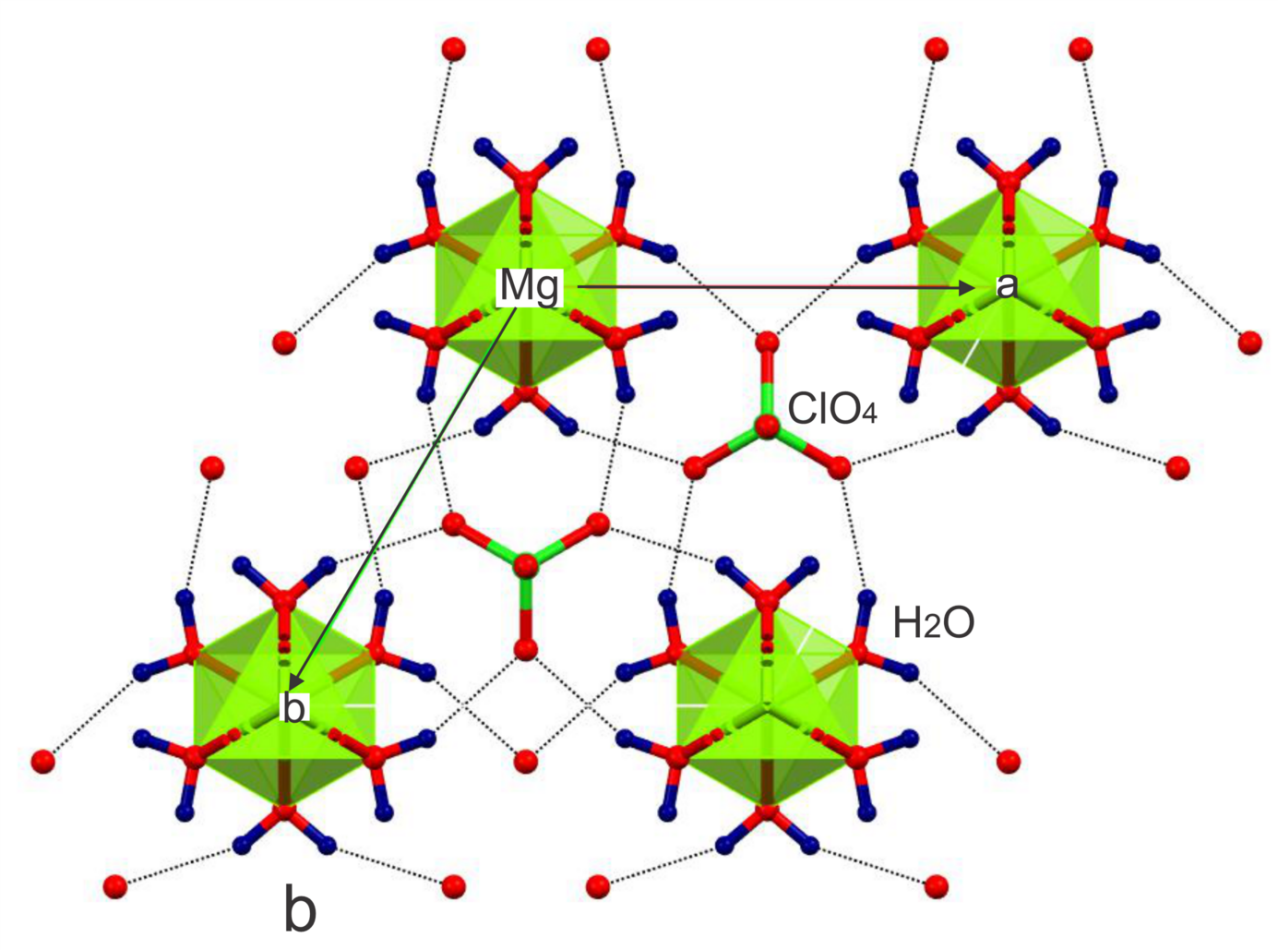
Packing of the crystal structure of Mg(ClO_4_)_2_·6H_2_O viewed along [001], illustrating the hydrogen-bonded framework and disordered octa­hedral chains.

The structure is consolidated by O1—H1⋯O3^i^ [symmetry code: (i) 1 − *x* + *y*, 1 − *x*, *z*) hydrogen bonds involving the coordinated water mol­ecule [H⋯O = 2.20 Å, O⋯O = 3.041 (4) Å, O—H⋯O = 155°], which link the coordination octa­hedra into a three-dimensional network. The arrangement of the octa­hedral chains and hydrogen-bond network viewed along the [001] direction is shown in Fig. 2 ▸.

## 4. Database survey

Previously reported crystal structures of magnesium perchlorate hydrates include ortho­rhom­bic and monoclinic polymorphs (West, 1934 ▸; Robertson & Bish, 2010 ▸; Solovyov, 2012 ▸). Related disordered perchlorate hexa­hydrates containing divalent metal cations such as Zn, Ni and Fe have also been described (Ghosh & Ray, 1976 ▸; Ghosh *et al.*, 1997 ▸). These compounds exhibit similar disorder-related structural features, including partial occupancies of the metal positions and disorder-driven symmetry relationships between hexa­gonal and ortho­rhom­bic forms.

## 5. Synthesis and crystallization

The compound was obtained in the system Mg(ClO_4_)_2_:*m*(acetamide):*n*(H_2_O) by solution crystallization followed by slow evaporation at room temperature. The mixture forms a viscous liquid phase.

Crystallization occurs over approximately two days, yielding large needle-like crystals suitable for single-crystal X-ray diffraction at early stages. Continued crystallization leads to deterioration in crystal quality and increased structural disorder.

## 6. Refinement

Crystal data, data collection and structure refinement details are summarized in Table 1 ▸. The model indicates the presence of disorder, as reflected in elevated displacement parameters and features identified in the *checkCIF* analysis. The hydrogen atom of the coordinated water mol­ecules was included in a calculated position and refined using a riding model.

## Supplementary Material

Crystal structure: contains datablock(s) I. DOI: 10.1107/S2056989026004871/hb8208sup1.cif

Structure factors: contains datablock(s) I. DOI: 10.1107/S2056989026004871/hb8208Isup2.hkl

CCDC reference: 2489227

Additional supporting information:  crystallographic information; 3D view; checkCIF report

## Figures and Tables

**Table 1:** Experimental details

Crystal data	
Chemical formula	Mg(ClO_4_)_2_·6H_2_O
*M*_r_	165.65
Crystal system, space group	Hexagonal, *P*6_3_*mc*
Temperature (K)	273
*a*, *c* (Å)	7.7942 (3), 5.2703 (3)
*V* (Å^3^)	277.27 (3)
*Z*	2
Radiation type	Mo *K*α
μ (mm^−1^)	0.72
Crystal size (mm)	0.2 × 0.1 × 0.1

Data collection	
Diffractometer	Bruker APEXII CCD
Absorption correction	Multi-scan (*SADABS*; Krause *et al.*, 2015 ▸)
*T*_min_, *T*_max_	0.870, 0.932
No. of measured, independent and observed [*I* > 2σ(*I*)] reflections	7335, 234, 229
*R*_int_	0.038
(sin θ/λ)_max_ (Å^−1^)	0.624

Refinement	
*R*[*F*^2^ > 2σ(*F*^2^)], *wR*(*F*^2^), *S*	0.024, 0.065, 1.20
No. of reflections	234
No. of parameters	22
No. of restraints	1
H-atom treatment	H-atom parameters constrained
Δρ_max_, Δρ_min_ (e Å^−3^)	0.15, −0.20
Absolute structure	Flack *x* determined using 98 quotients [(*I*^+^)−(*I*^−^)]/[(*I*^+^)+(*I*^−^)] (Parsons et al., 2013)
Absolute structure parameter	0.04 (3)

Computer programs: *APEX2* and *SAINT* (Bruker, 2016 ▸), *SHELXT2018/2* (Sheldrick, 2015*a* ▸), *SHELXL2018/3* (Sheldrick, 2015*b* ▸) and *OLEX2* (Dolomanov *et al.*, 2009 ▸).

## supplementary crystallographic information

### Magnesium perchlorate hexahydrate Crystal data

**Table:**

Mg(ClO_4_)_2_·6H_2_O	*D*_x_ = 1.984 Mg m^−^^3^
*M**_r_* = 165.65	Mo *K*α radiation, λ = 0.71073 Å
Hexagonal, *P*6_3_*m**c*	Cell parameters from 4768 reflections
*a* = 7.7942 (3) Å	θ = 3.0–25.9°
*c* = 5.2703 (3) Å	µ = 0.72 mm^−^^1^
*V* = 277.27 (3) Å^3^	*T* = 273 K
*Z* = 2	Block, clear white
*F*(000) = 170	0.2 × 0.1 × 0.1 mm

### Magnesium perchlorate hexahydrate Data collection

**Table:**

Bruker APEXII CCD diffractometer	229 reflections with *I* > 2σ(*I*)
Radiation source: sealed tube	*R*_int_ = 0.038
φ and ω scans	θ_max_ = 26.4°, θ_min_ = 3.0°
Absorption correction: multi-scan (SADABS; Krause *et al.*, 2015)	*h* = −9→9
*T*_min_ = 0.870, *T*_max_ = 0.932	*k* = −9→9
7335 measured reflections	*l* = −6→6
234 independent reflections	

### Magnesium perchlorate hexahydrate Refinement

**Table:**

Refinement on *F*^2^	Secondary atom site location: difference Fourier map
Least-squares matrix: full	Hydrogen site location: difference Fourier map
*R*[*F*^2^ > 2σ(*F*^2^)] = 0.024	H-atom parameters constrained
*wR*(*F*^2^) = 0.065	*w* = 1/[σ^2^(*F*_o_^2^) + (0.0391*P*)^2^ + 0.061*P*] where *P* = (*F*_o_^2^ + 2*F*_c_^2^)/3
*S* = 1.20	(Δ/σ)_max_ < 0.001
234 reflections	Δρ_max_ = 0.15 e Å^−^^3^
22 parameters	Δρ_min_ = −0.20 e Å^−^^3^
1 restraint	Absolute structure: Flack *x* determined using 98 quotients [(*I*^+^)-(*I*^-^)]/[(*I*^+^)+(*I*^-^)] (Parsons et al., 2013)
Primary atom site location: structure-invariant direct methods	Absolute structure parameter: 0.04 (3)

### Magnesium perchlorate hexahydrate Special details

**Table:**

Geometry. All esds (except the esd in the dihedral angle between two l.s. planes) are estimated using the full covariance matrix. The cell esds are taken into account individually in the estimation of esds in distances, angles and torsion angles; correlations between esds in cell parameters are only used when they are defined by crystal symmetry. An approximate (isotropic) treatment of cell esds is used for estimating esds involving l.s. planes.

### Magnesium perchlorate hexahydrate Fractional atomic coordinates and isotropic or equivalent isotropic displacement parameters (Å^2^)

**Table:**

	*x*	*y*	*z*	*U*_iso_*/*U*_eq_	Occ. (<1)
Mg1	0.000000	0.000000	0.2498 (12)	0.0239 (7)	0.5
O1	0.12365 (19)	−0.12365 (19)	0.5000 (9)	0.0467 (7)	
H1	0.255441	−0.078800	0.499143	0.070*	
Cl1	0.666667	0.333333	0.49460 (13)	0.0291 (4)	
O2	0.666667	0.333333	0.2241 (10)	0.0576 (17)	
O3	0.8658 (5)	0.4329 (2)	0.5864 (8)	0.0579 (10)	

### Magnesium perchlorate hexahydrate Atomic displacement parameters (Å^2^)

**Table:**

	*U*^11^	*U*^22^	*U*^33^	*U*^12^	*U*^13^	*U*^23^
Mg1	0.0220 (9)	0.0220 (9)	0.0277 (12)	0.0110 (4)	0.000	0.000
O1	0.0418 (10)	0.0418 (10)	0.0639 (14)	0.0264 (11)	0.0007 (9)	−0.0007 (9)
Cl1	0.0237 (5)	0.0237 (5)	0.0400 (6)	0.0118 (2)	0.000	0.000
O2	0.067 (3)	0.067 (3)	0.040 (3)	0.0333 (13)	0.000	0.000
O3	0.0294 (16)	0.0534 (15)	0.083 (2)	0.0147 (8)	−0.0159 (15)	−0.0079 (8)

### Magnesium perchlorate hexahydrate Geometric parameters (Å, º)

**Table:**

Mg1—Mg1^i^	2.6352 (1)	Mg1—O1^vi^	2.126 (5)
Mg1—Mg1^ii^	2.6351 (1)	O1—H1	0.9046
Mg1—O1^i^	2.126 (5)	O1—H1^vii^	0.9046
Mg1—O1	2.127 (5)	Cl1—O2	1.426 (5)
Mg1—O1^iii^	2.127 (5)	Cl1—O3	1.428 (3)
Mg1—O1^iv^	2.126 (5)	Cl1—O3^viii^	1.428 (3)
Mg1—O1^v^	2.127 (5)	Cl1—O3^ix^	1.428 (3)
			
O1^i^—Mg1—O1^v^	94.35 (6)	O1^iv^—Mg1—O1^iii^	94.35 (6)
O1^vi^—Mg1—O1^iii^	94.35 (6)	Mg1^ii^—O1—Mg1	76.57 (9)
O1^vi^—Mg1—O1^i^	85.7 (2)	Mg1^ii^—O1—H1^vii^	120.83 (7)
O1^i^—Mg1—O1	94.35 (6)	Mg1^ii^—O1—H1	120.8
O1^iv^—Mg1—O1	94.35 (6)	Mg1—O1—H1^vii^	120.39 (7)
O1^vi^—Mg1—O1^v^	94.35 (6)	Mg1—O1—H1	120.4
O1^iv^—Mg1—O1^i^	85.7 (2)	H1—O1—H1^vii^	99.1
O1—Mg1—O1^v^	85.6 (2)	O2—Cl1—O3^viii^	109.79 (19)
O1^i^—Mg1—O1^iii^	179.9 (3)	O2—Cl1—O3	109.79 (19)
O1^iv^—Mg1—O1^v^	179.9 (3)	O2—Cl1—O3^ix^	109.79 (19)
O1^vi^—Mg1—O1	179.9 (3)	O3—Cl1—O3^viii^	109.15 (19)
O1^iv^—Mg1—O1^vi^	85.7 (2)	O3^ix^—Cl1—O3	109.15 (19)
O1^v^—Mg1—O1^iii^	85.6 (2)	O3^ix^—Cl1—O3^viii^	109.15 (19)
O1—Mg1—O1^iii^	85.6 (2)		

Symmetry codes: (i) *x*−*y*, *x*, *z*−1/2; (ii) *x*−*y*, *x*, *z*+1/2; (iii) −*x*+*y*, −*x*, *z*; (iv) *y*, −*x*+*y*, *z*−1/2; (v) −*y*, *x*−*y*, *z*; (vi) −*x*, −*y*, *z*−1/2; (vii) −*y*, −*x*, *z*; (viii) −*x*+*y*+1, −*x*+1, *z*; (ix) −*y*+1, *x*−*y*, *z*.
